# Feasibility of Oil Spill Detection in Port Environments Based on UV Imagery

**DOI:** 10.3390/s25061927

**Published:** 2025-03-20

**Authors:** Marian-Daniel Iordache, Françoise Viallefont-Robinet, Gert Strackx, Lisa Landuyt, Robrecht Moelans, Dirk Nuyts, Joeri Vandeperre, Els Knaeps

**Affiliations:** 1 Remote Sensing Department, Flemish Institute for Technological Research (VITO), Boeretang 200, 2400 Mol, Belgium; gert.strackx@vito.be (G.S.); lisa.landuyt@vito.be (L.L.); robrecht.moelans@vito.be (R.M.); dirk.nuyts@vito.be (D.N.); els.knaeps@vito.be (E.K.); 2 DOTA, ONERA, Université de Toulouse, 31000 Toulouse, France; francoise.viallefont@onera.fr; 3 Port of Antwerp-Bruges (POAB), Zaha Hadidplein 1, 2030 Antwerp, Belgium; joeri.vandeperre@portofantwerpbruges.com

**Keywords:** oil spill, ultraviolet imagery, class separability, environmental pollution, monitoring

## Abstract

Oil spills in ports are particular cases of oil pollution in water environments that call for specific monitoring measures. Apart from the ecological threats that they pose, their proximity to human activities and the financial losses induced by disturbed port activities add to the need for immediate action. However, in ports, established methods based on short-wave infrared sensors might not be applicable due to the relatively low thickness of the oil layer, and satellite images suffer from insufficient spatial resolution, given the agglomeration of objects in ports. In this study, a lightweight ultraviolet (UV) camera was exploited in both controlled experiments and a real port environment to estimate the potential and limitations of UV imagery in detecting oil spills, in comparison to RGB images. Specifically, motivated by the scarce research literature on this topic, we set up experiments simulating oil spills with various oil types, different viewing angles, and under different weather conditions, such that the separability between oil and background (water) could be better understood and objectively assessed. The UV camera was also used to detect real-world oil spills in a port environment after installing it on a vessel for continuous monitoring. Various separability metrics between water and oil, computed in both scenarios (controlled experiments and port environment), show that the UV cameras have better potential than RGB in detecting oil spills in port environments.

## 1. Introduction

Oil spills pose threats to ecosystem balance and human health. They constitute emergency situations in which the correct identification of the affected area and the prompt mitigation measures is critical to avoid environmental damage and financial losses. At sea, the largest spills in terms of volume happen at oil extraction sites, where they can be dramatically larger than any spill involving tankers. For example, it is estimated that the explosion at the Deepwater Horizon mobile drilling rig on 20 April 2010 caused around 700,000 tons of crude oil to be released into the Gulf of Mexico [1,2]. When caused by a tanker, a spill is considered to be large if it exceeds 700 tons, as indicated in [3], where the authors also mentioned that only one such spill occurred worldwide in 2023. Very large spills are thus events that occur with low frequency, but their potential environmental damage is devastating, as they are harmful to marine birds, mammals, fish, and shellfish, among others, impacting not only the ecosystem but also parts of the food chain and human food resources [4]. In many cases, the oil forms so-called oil slicks due to its lower density, i.e., continuous films on the water surface; however, heavy oils could also sink if evaporation of the lighter compounds occurs, meaning that organisms from all water layers and the bottom surface would be at risk. For birds and mammals, the oil impacts the insulating and water-repellent abilities of fur and feathers, leaving them exposed to harsh elements and leading to their death due to hypothermia [5]. Oil pollution affects the health and reproductive capacity of fish, birds (pelicans, gulls, and many others), sea turtles, and mammals (pinnipeds, cetaceans, manatees, sea otters, polar bears, dolphins, whales), but it also impacts invertebrates and plants [5,6]. Apart from their dramatic impact on the ecosystem, oil spills also have economic implications, not only short-term ones related to the cleanup activities, but also in the medium and long term, e.g., by making the exploitation of natural resources through fishing impossible. The cost of cleaning an oil spill is difficult to model and can vary significantly, as it depends on a multitude of specific traits, such as oil type (chemical composition), quantity, viscosity, tendency to emulsify, environmental conditions (temperature, wind, waves), weathering, spill location, etc., and many models and algorithms are inherently limited as they are based on empirical studies [7]. For specific cases, the cleaning cost estimates indicate that the financial losses can be very high: a ship that ran aground in New Zealand in 2011 released more than 350 tons of heavy fuel oil and other contaminants, leading to a conservative cleaning cost of 60–70 million USD [8], and the Deepwater Horizon accident cost the exploiting company around 69 billion USD, of which approximately 14 billion USD was committed only for response and cleaning activities, according to their own reports [9].

Given the dramatic consequences of oil spills, intensive research efforts have been devoted to mitigating their effects. A critical part of such efforts is the detection and accurate delineation of the spill’s extent, which allow for rapid intervention in containing the spread and removing the spilled substances from the area. Remote sensing methods have been extensively employed in recent years to test their capabilities in observing and delimiting spills in open water. Both passive and active sensors are used to detect oil spills. The former are sensors that sense radiation that is naturally reflected or emitted by the observed objects, while the latter excite the observed surface using a source of radiation (light) and then sense the response of the observed object to this radiation. Optical imagery can be useful to distinguish oil from other materials (algae, water). In the UV spectral region, one of the most widespread techniques when dealing with crude oil is based on laser fluorosensors, which are active systems using a UV laser to excite oil compounds such that they emit radiation at higher wavelengths due to their fluorescent properties. The sensor is then able to observe the difference in the quantities of radiation received from the polluted and the clean areas [10,11,12,13], even at night. Due to the use of additional equipment (UV lasers), such a system is heavy and expensive. In some works, the use of passive UV sensors for oil spill detection, based on the contrast between the spill and water, has been investigated for crude oil based on airborne hyperspectral sensors [14,15,16] and spaceborne multispectral sensors [17,18]. In the visible range, non-emulsified oil has no specific spectral features, which impedes its straightforward discrimination, along with the influence of other factors (illumination-view geometry, oil condition and thickness, weather) [19,20,21,22]. Multispectral sensors carried by satellite platforms such as Sentinel-2, MODIS, or Landsat have also been exploited for oil spill detection (see [19] and the references therein). Hyperspectral data (airborne, spaceborne, or point measurements) benefit from high spectral resolution, thus being able to capture fine features in the short-wave infrared (SWIR) range, which can be helpful in distinguishing between oil and other materials, and even between different types of oil [23,24,25,26,27,28]. However, they require intensive data preprocessing, and they are more expensive to obtain than other imagery types, especially when real-time monitoring is targeted [29]. Thermal imagery in the long-wave infrared spectral region (8–14 µm) has proven to be useful in detecting thick oil spills (>500 µm), but it is not useful to detect thinner oil layers [30,31]. A consistent number of scientific papers deal with oil spills by exploiting Synthetic-Aperture Radar (SAR) images [32,33,34,35]. SAR images offer advantages over optical images: they can operate during both day and night, they penetrate through clouds and smoke, and they are not affected by sun glint on water areas, among other factors. The principle behind spotting oil spills on water based on SAR imagery is that the oil film reduces the backscattering of the water area as it attenuates the roughness of the surface; thus, the affected area appears darker than the surrounding water (see [36] and the references therein).

In this paper, a special case of oil spills is under investigation: oil spills occurring in port environments. In this type of environment, at least locally, the water’s surface roughness may not be high enough to be sensitive to an oil spill, preventing full efficiency of SAR-based methods. Oil spills in ports have characteristics that differ from those happening at sea or on shorelines, impeding the application of many methods developed for open-sea spills. First, it is usually refined oil and not crude oil, as is the case for extraction platforms and large tankers. Second, the thickness of the oil is low, meaning that methods based on thermal images or SWIR data are not efficient. Third, ports are crowded places in which fixed constructions and mobile objects are present, and spaceborne imagery, either optical or SAR, is less efficient due to the relatively coarse spatial resolution. A very important characteristic is that, unlike accidents happening at sea, large numbers of people are usually present in ports (port employees, clients, visitors). Oil spills in ports are events that occur with relatively high frequency. Even spills in low quantities, e.g., leaks during the refueling of ship tanks, should be treated seriously, as their harmful effects on the environment are certain. Furthermore, a large number of studies show that prolonged exposure to oil compounds could induce a variety of diseases in humans, including increased risk of cancer [37,38,39,40]. This means that spilled oil should be cleaned as soon as possible, such that port workers are minimally exposed. Furthermore, fast intervention is also needed from an economic point of view—when a spill occurs, financial losses are induced not only by the cleaning operations but also by the interruption or disturbance of the port’s activities. Ideally, a solution should be designed based on lightweight sensors that can be operated at the port from mobile platforms (e.g., drones or vessels), at low cost, and with very low risk of injuries or physical damage in case of accidents.

As mentioned above, many of the available techniques are either not applicable or have not been tested for the specific traits of spills occurring in port environments. However, for thin oil films, lightweight passive sensors in the ultraviolet (UV) spectral region, underdeveloped as compared to other methods [14], could be useful in delimiting the spill. Specifically, the oil–water contrast is usually positive due to the higher refractive index of the oil in comparison to water, such that the oil reflects more incoming radiation than the surrounding water (see seos-project.eu; last accessed: 5 September 2024). However, positive contrast may occur not only for oil but also for cloud reflection. Moreover, another physical phenomenon, linked to the surface roughness, is in competition with the effect of the refractive index difference. Indeed, if oil dampens the surface roughness enough, outside the specular direction, the scattered signal is higher on water than on oil. The surface roughness effect leads to a negative oil–water contrast. Such a contrast can also occur between wind slicks and water. Nevertheless, a refractive index effect leading to a positive oil–water contrast is assumed for ports.

In this paper, the use of passive UV sensors for oil spill detection in port environments is investigated, motivated by the expected behavior based on physical phenomena and the identified lack of in-depth studies on this subject. Specifically, this study uses an experimental approach to shed light on the following fundamental questions: (i) Can passive UV sensors sense oil spills under the circumstances occurring in ports? (ii) Under which viewing and environmental conditions is it possible to detect oil spills by using passive UV sensors? (iii) What are the advantages of passive UV sensors over RGB sensors? (iv) Are the findings found in controlled environments applicable to real-world images? This study is the first to apply a twofold experimental approach to tackle these questions. First, the experiments were designed in a controlled environment to exploit the capabilities of a passive UV sensor to observe oil spills from various viewing angles and environmental conditions. Statistical metrics were computed in order to analyze the separability between the oil and the water areas under various experimental configurations. Second, the same sensing camera was installed on a vessel in a real port environment to analyze whether the experimental findings derived under controlled conditions would be confirmed by the observations made from the vessel during real spill events.

The rest of this paper is organized as follows: Section 2 describes the experimental setup. Section 3 presents the findings extracted from the performed experiments, followed by a discussion in Section 4. Finally, Section 5 draws the overall conclusions and includes pointers for future work.

## 2. Materials and Methods

Hereafter, for simplicity, the terms “UV sensor” and “UV camera” refer to the “passive UV sensor” and “passive UV camera”, respectively. The main goal of the experiments described in this paper was to objectively assess the potential and limitations of such sensors in dealing with oil spills under the specific conditions encountered in ports. In order to achieve this goal, a twofold approach was followed:

(1) First, outdoor controlled experiments were designed such that various parameters varied; some of these parameters were controlled (oil type, viewing angle), while others were not (environmental conditions).
(2) Once the controlled experiments were concluded, the UV camera was installed on a cleaning vessel at the Port of Antwerp–Bruges for permanent data acquisition (during daylight).

In both cases, RGB cameras were also used to scan the water area to allow for comparisons between the two sensor types. Simple statistical metrics were then used to assess the separability between the water and oil areas.

### 2.1. UV Camera Characterization

**Figure 1:**
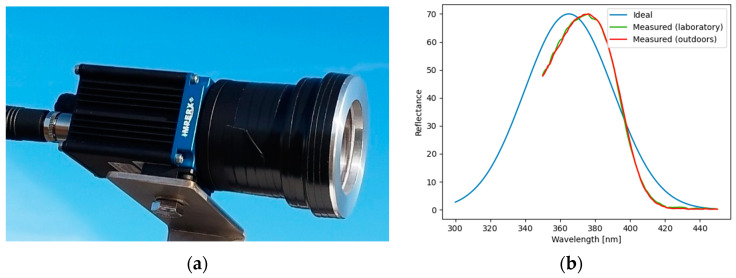
UV camera used in the experiments: (**a**) External view. (**b**) UV filter response (blue—provided by the producer; green—measured in a laboratory; red—measured outdoors).

A ruggedized camera, resistant to weather elements (IP67 rating), was used in the experiments. A UV filter was acquired separately and mounted in front of the camera lens. The characteristics of the filter were investigated under various conditions before acquiring the oil spill imagery. Figure 1a shows a picture of the camera. According to the specifications, the UV filter has a central wavelength of 365 nm and a full width at half-maximum (FWHM) of 60 nm. During the camera tests, the reflectance of a reference spectralon, observed through the UV filter, was acquired with an ASD spectrometer operating in the spectral range 350–2500 nm, with a spectral sampling of 1 nm, under two conditions: (i) in a dark room dedicated to spectral measurements at VITO premises, and (ii) outdoors, on a cloudy day. The ideal response function and those measured by the ASD spectrometer are shown in Figure 1b. It can be seen that the shape of the response function resembles the Gaussian shape; however, the central wavelength is shifted by approximately 10 nm towards the blue region of the electromagnetic spectrum. However, this filter can be considered appropriate for our experiments, as it is sensitive in the desired spectral region (below 400 nm).

The camera acquires images with 2848 × 2856 pixels and has a measured field of view of 16.2°. Due to these characteristics, the spatial resolution of the camera is high, e.g., for distances lower than 5 m between the camera and the target, the spatial resolution (assimilated to the ground sampling distance) is better than 1 mm. The camera software allows for the variation in data acquisition parameters such as gains and integration times. The acquired images are stored in .tif format.

### 2.2. Controlled Experiments

#### 2.2.1. Experimental Site and Setup

**Figure 2:**
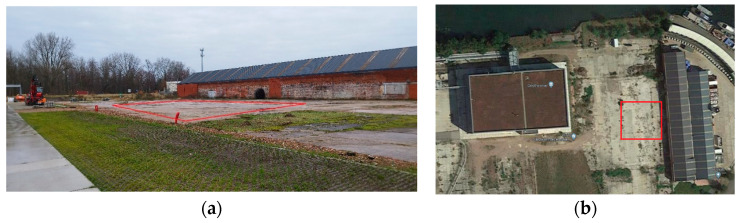
Selected site for the controlled experiments: (**a**) Picture of the selected site. (**b**) Aerial view (Google Maps) of the selected site. The red box indicates the approximate area used during the experiments.

An empty parking at VITO premises (Mol, Belgium; geographic coordinates: 51°13′27′′ N, 5°06′44′′ E) was selected as the experimental site. Figure 2a shows a picture taken on-site, in which the red rectangle delimits the area reserved for the experiments, and Figure 2b shows a Google Maps view of the site.

A high-resolution RLC-1212A RGB camera (see https://reolink.com/product/rlc-1212a/?srsltid=AfmBOoraSV_9Q3Utrp7JUvG2JlnfWoa2JwZ9_tIJdTidFCUbEabMrPBc; last accessed: 20 September 2024) was installed next to the UV camera for comparison purposes. The RGB camera recorded video files with a framerate of 2fps, and it was operated in automatic exposure mode. For the analysis presented in this paper, individual frames were extracted and treated as separated images.

**Figure 3:**
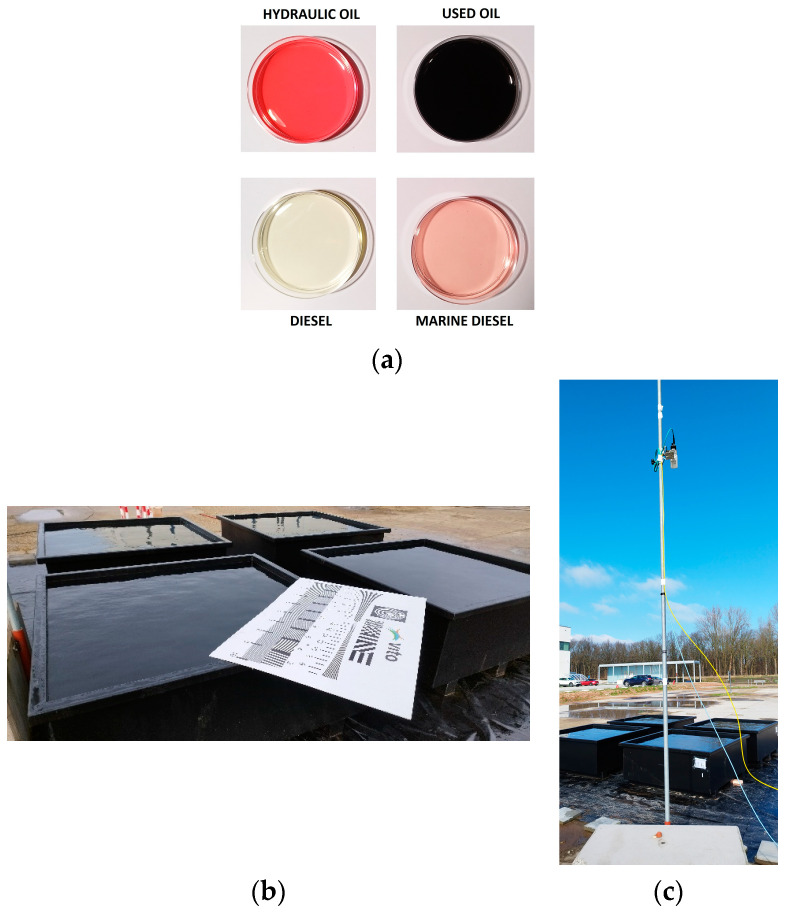
Oil samples and site arrangement: (**a**) Pictures of the four oil samples. (**b**) Metallic containers. (**c**) UV and RGB cameras mounted on a common pole.

Four refined oil types were considered in the experiments: hydraulic oil (H), used oil (U), diesel (D), and marine diesel (M). Samples of the oils are shown in Figure 3a. These samples were photographed in transparent plastic receptacles placed on white printing paper, illuminated from an oblique angle by two lateral lamps, and with an oil layer thickness of approximately 5 mm. The oils were provided by the Port of Antwerp–Bruges, and they are commonly used in the machinery operating in the port. For each oil type, a metallic container of 1.5 m × 1.5 m × 0.5 m in size was built. The four containers were placed over plastic foil such that potential accidental spills during the experiments were contained. A procedure ensuring the proper handling and treatment of the polluted water was designed in consultation with specialized environmental protection teams and was followed strictly. A distance of 40 cm was kept between the containers in all directions, for easy access. The cameras were mounted on a common platform (a metallic pole) to acquire concomitant imagery. The pole was installed on a mobile platform, allowing us to vary the observation angles and distances. Three observation angles were considered: near-nadir (NN), 45°, and 30°. The angles were measured between the horizontal line and the hypothetical line from the camera to the center of the water surface. The camera positions were manually adjusted to obtain the predefined angles by using precise instruments (protractors and rulers). Note that we use a near-nadir view instead of a nadir view, as the pole could not be placed exactly at the center of the water’s surface. In practical scenarios, where a platform carries the cameras at the port, nadir or near-nadir views are not encouraged, as the field of view diminishes considerably as compared to other angles. They were still important in the controlled experiments, as they can reveal potential differences between different viewing angles when solving oil spill delineation tasks. The setup is illustrated in Figure 3b–c.

#### 2.2.2. Data Acquisition

Three series of measurements were performed on the 6, 20, and 27 February 2024. The weather conditions varied between the dates and even during the same day. While this can be seen as a disadvantage when comparing different scenarios, it is also beneficial, as it allows for more diverse scenarios—e.g., sun glint effects can be observed under sunny weather, but not in cloudy weather. In this study, without loss of generality, only the data from the last campaign were analyzed, due to their superior quality. However, the data from 20 February were used to a small extent, as shown in Section 3.3, to better understand the effects of weather conditions on the acquired imagery.

The oil spills were simulated by manually dropping oil on the water’s surface. Relatively low quantities of oil were used during the measurements. The typical quantity to start with was 0.1 mL for the NN view, and then 0.1 mL was added for the 45° view, and 0.2 mL was added for the 30° view. The oil quantities were measured by low dead-volume (LDV) syringes. The UV images were taken by manually triggering the image capture option in the data acquisition software. RGB images (frames extracted from the videos) and UV images were then paired according to the minimal timestamp difference. It should be noted that the UV camera settings (fixed gain and integration time) and the RGB camera settings (automatic auto-exposure mode) were kept constant across the measurements.

Table 1 details the measurements under analysis, along with the environmental conditions. Each elementary experiment has a unique identifier, as shown in the second column of the table. Overall, the NN images were taken under cloudy weather earlier in the morning, while the others were acquired under mixed or clear skies around noon and in the afternoon (local time). Regarding the wind speed, “regular wind” indicates speeds of 10–12 km/h, and “mild wind” indicates speeds of 7–9 km/h. During the experiment denominated “H45_R”, the cameras with a viewing angle of 45° were moved around the container reserved for hydraulic oil spills, such that 6 scanning positions covered a sector of a circle of 200°, with the first position having the Sun at the back of the cameras. This experiment was intended to reveal how the relative viewing azimuth (difference between the viewing azimuth and sun azimuth) influences the visibility of the oil.

#### 2.2.3. Data Preprocessing

As previously mentioned, the RGB measurements are, in practice, low-framerate movies acquired during the experiments. All of the frames of these movies were extracted to individual files, and their respective timestamps were computed such that they could be paired with UV images. In the following sections, all references to RGB data point to these images. The pairs of RGB and UV images were created based on the timestamps. Exact matching of the images with respect to acquisition time was not possible, as the video framerate was finite, but time differences lower than half a second were deemed satisfactory. From all available pairs, the ones containing objects or persons were manually removed.

**Figure 4:**
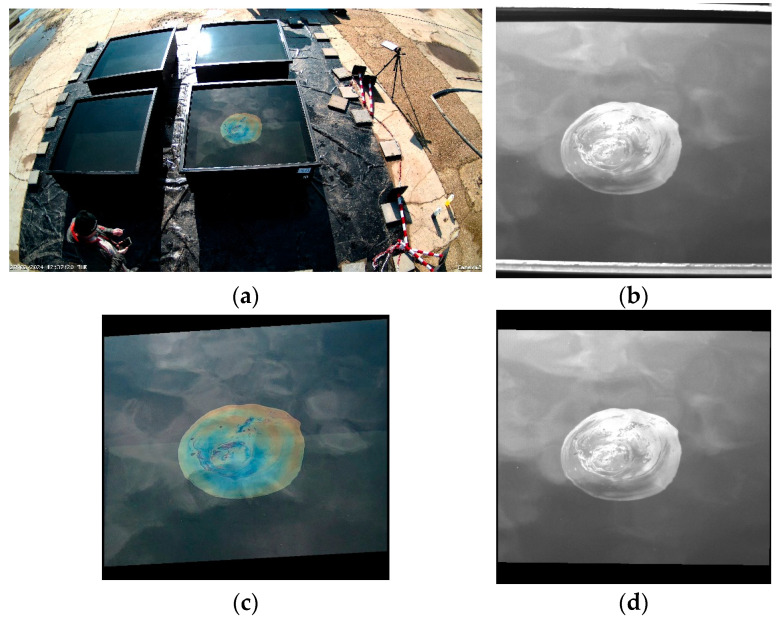
Example of original and cropped images used in comparisons: (**a**) original RGB image; (**b**) original UV image; (**c**) cropped RGB image; (**d**) cropped UV image.

Figure 4a,b show a pair of RGB-UV images obtained during the D_45 experiment. For a fair comparison, the images were manually cropped so that they covered approximately the same area, as seen in Figure 4c,d. Note that the cropping also removed parts falling outside the water area, e.g., container edges. Due to the manual adjustment of the camera positions, the slightly different timestamps between the RGB and UV images, and in some cases the lack of correspondent fixed points between paired RGB and UV images, no universal homography matrix could be defined to ensure the alignment of the image pairs through reprojections. Despite this inconvenience, a statistical analysis of the cropped images could be performed without loss of critical details, given that series of image pairs were used for each of the experiments. For each experiment, a set of at least three image pairs was randomly selected, and the oil and water areas were manually drawn. The pixels contained by these areas were then used as inputs to compute the statistical metrics described in Section 2.4.

### 2.3. Data Acquisition in a Real Port Environment

#### 2.3.1. Data Acquisition System

**Figure 5:**
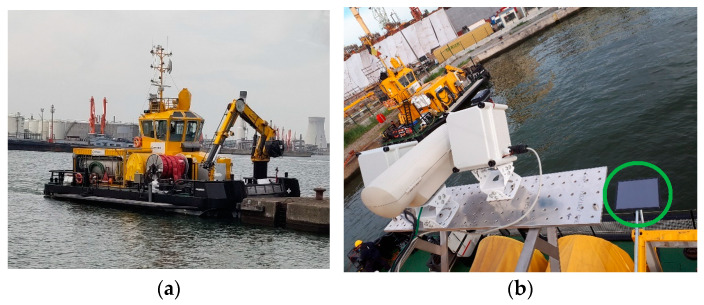
Setup of real-world data acquisition system: (**a**) The vessel PROGRESS hosting the data acquisition system. (**b**) Platform able to accommodate a multi-camera system.

After the completion of the controlled experiments, the UV and RGB cameras were mounted on a platform onboard the PROGRESS vessel at the Port of Antwerp–Bruges, next to other instruments. PROGRESS is a vessel owned by Brabo Group Antwerp, a Multicat 1908 Utility vessel used for oil spill cleanup in the Port of Antwerp–Bruges, among other activities. On its starboard side, PROGRESS is equipped with an oil boom sweeping system and a work platform. In case of an oil spill incident, this is the side where all of the actions take place, so the camera system points to that part of the water surface. The camera system’s recordings were triggered based on a schedule and activity estimation. The recorded information was automatically uploaded to the VITO data processing facility. While docked or during cleanup activities, the system was monitored, managed, and updated remotely via the mobile network (Wi-Fi or 4G/5G) to avoid on-site interventions as much as possible. Figure 5 shows the PROGRESS vessel and the common installation platform for the cameras. Each camera had its own 3-axis pointing mount to carefully adjust the viewing direction. In the reported experiments, the viewing angle, measured between the camera axis and the vertical direction, was fixed to approximately 40°. The cameras were placed inside individual weatherproof enclosures. Data were acquired for three months (March–May 2024), resulting in a dataset of over 200 GB.

In Figure 5b, the green circle indicates a calibration panel that lays partially in the field of view of the cameras. This panel does not have special spectral properties except for a uniform reflectance across its entire surface, acting as a constant that can be used to account for variability in illumination, as shown below.

#### 2.3.2. Image Preprocessing

**Figure 6:**
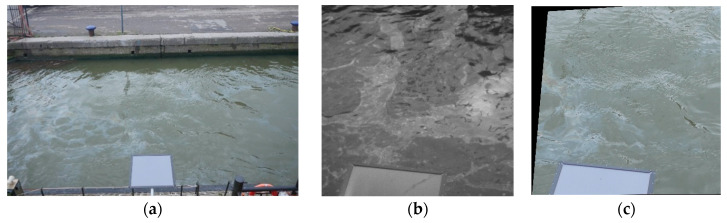
Examples of RGB and UV images acquired by the data acquisition system installed onboard the cleaning vessel: (**a**) Example of an RGB image. (**b**) Example of corresponding UV image. (**c**) Preprocessed RGB image.

A pair of RGB-UV images is shown in Figure 6a,b. Note that the RGB camera has a wider field of view in comparison to the UV camera. It can also be seen that the two images are rotated with respect to each other, and the viewing angles are different. An alignment of the images is needed to perform fair comparisons between the capabilities of the two cameras. The presence of the calibration panel in all images ensures that there are enough common reference points to perform an image co-registration between the UV and RGB images. It was determined that the two cameras provide images that are rotated with an angle of approximately 8.21823 degrees with respect to each other. First, this angle was used to rotate the RGB image to better align with the UV image. Second, a resampling of the RGB image was performed such that the spatial resolutions of the compared images were similar. Third, the RGB image was cropped to cover approximately the same area as the UV image. Finally, a co-registration procedure based on manually defined key points was applied to warp the RGB image such that the two images could benefit from common observation angles. In this way, RGB and UV images with similar fields of view, viewing angles, and spatial resolutions were obtained for further comparisons. Figure 6c shows the RGB image obtained after preprocessing the image from Figure 6a. In the actual comparisons, only subsets containing oil and water were compared by selecting suitable areas that did not overlap with the reference panel.

The three bands of the RGB images were extracted to separate the files. A gray version of the RGB images was also generated using the functionalities of Python’s scikit-image package [41], based on the following formula: Y = 0.2125 R + 0.7154 G + 0.0721 B, where Y is the gray image obtained and R, G, and B are the intensities in the red, green, and blue channels, respectively. All of these images were later used in comparisons with the corresponding UV images.

It is important to note that the UV camera acquired images with a variety of settings, which in the long term provided sufficient data to derive optimal acquisition parameters. Specifically, the gains and the integration times were varied during imaging, as follows:

- Gains: 20 and 30.
- Integration times (ITs): 1, 2, 5, 8, 10, 20, 30, 40, and 60 ms.

The cameras installed onboard the vessel took turns acquiring imagery, meaning that there were no exact timestamp matches. Specifically, two RGB images were taken approximately every 15 min. Immediately after the second RGB image was recorded, a series of UV images was recorded. This series included all of the combinations of considered gains and integration times, for a total of 18 images. Given that some combinations did not ensure good data quality, there could be a difference of tens of seconds between an RGB image and a UV image with good quality. The UV image acquisition was performed in less than one minute; thus, a larger time gap (approximately 14 min) occurred between the UV image and the next RGB image. Moreover, the vessel was in continuous motion and changed direction, while the shape of the oil spill also varied; thus, it was not guaranteed that the oil would be captured in consecutive images, leading to an increased time gap between consecutive images containing oil. For example, the time difference between the RGB and UV images from Figure 6a,b is approximately 44 min. However, a statistical analysis is still possible by considering all of the images of oil spills acquired during the same day as separate instances of the same spill.

#### 2.3.3. Estimation of Optimal Camera Parameters

As mentioned above, the UV camera onboard the PROGRESS vessel recorded series of images with different camera settings. These can be seen as batches of images, allowing for data quality evaluations. Lower gains and integration times are likely to be insufficient to record a good signal, especially in low-illumination conditions, while higher gains and integration times might lead to image saturation. However, the inclusion of the reference panel in the field of view allows for a normalization of the images, which alleviates the pixel intensity differences. Once normalized images are obtained, simple methods to assess their quality can be employed, e.g., counting zero-valued and saturated pixels, and the images with the highest quality indicate the optimal ranges of gains and integration times that should be used for image acquisition. The procedure is demonstrated for data acquired from the PROGRESS vessel later in this paper, in Section 3.2.2.

### 2.4. Discrimination Metrics

**Figure 7:**
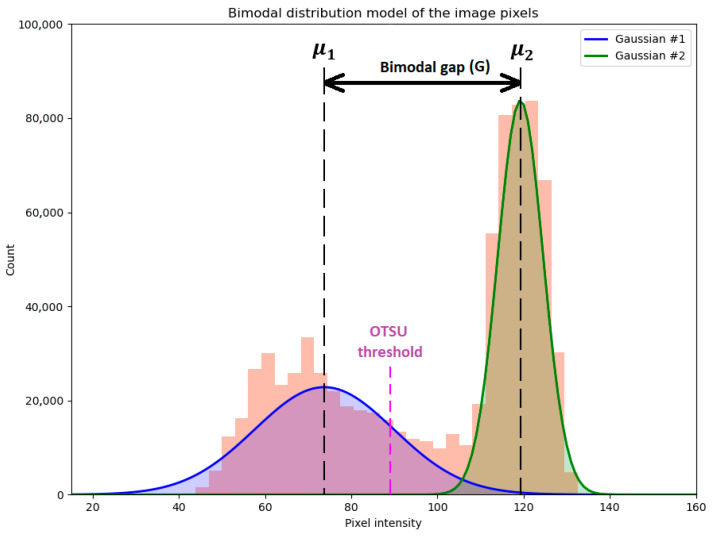
Illustration of the class separability assessment via bimodal distributions of image histograms.

The main criterion in which we were interested was the class separability between the classes “water” and “oil”. Note that other classes might be contained in an image; however, the cropped images included in the analysis only contained water areas partially polluted by oil, which served the overall goal of comparing the data types purely from a class separability point of view. Areas containing invalid values, such as saturated pixels, were neglected. Figure 7 illustrates the separability principle based on image histograms. For any image, the corresponding histogram can be viewed as a multi-modal Gaussian distribution, where the number of modes equals the number of pixel classes. Under the assumption that the analyzed images only contain water and oil areas, the distribution model becomes bimodal. The metrics used to assess the separability between classes are described below. All of the metrics are reported later in this paper as percentage differences relative to the best-found value, measured in absolute terms:

(1) $$R_{diff}=\frac{\left|V_{best}-V_{c}\right|}{V_{best}}\cdot 100,$$

where $R_{diff}$ is the computed value, $V_{best}$ is the best-found value across all data types (bands), and $V_{c}$ is the computed metric value for the current data type (band). This approach is preferred because absolute values or differences are less informative due to the expected differences between the pixel intensities acquired for different oil types over water.

→ Metric #1: Bimodal gap (*G*)

A better separability is obtained when the Gaussian means are placed as far as possible; thus, the so-called “bimodal gap” *G* in Figure 7, which measures the distance between the mean values of the Gaussian distributions, is large (and the standard deviations are ideally low). A small gap indicates higher confusion between the two classes and, thus, higher difficulty in distinguishing between them. Thus, the bimodal gap is one of the metrics used to assess the classes’ separability:

(2) $$G=\mu_{2}-\mu_{1},$$

where G denotes the gap, and $\mu_{1}$ and $\mu_{2}$ are the means of the two Gaussian distributions.

→ Metric #2: Normalized between-class variance (*η*)

When dealing with class separation based on thresholding, a well-established method is OTSU thresholding [42,43,44]. The algorithm provides an optimal value to separate the image parts based on their intensity values, which is useful, e.g., to distinguish between background and foreground pixels. In the context of oil spills, the clean water parts can be considered as the background, and the polluted parts constitute the foreground. The value of the threshold itself is not indicative of the separability of the classes; however, the OTSU threshold is derived by maximizing the inter-class variance *η*. A simple rule indicates that the class separability is higher when *η* is higher. In this work, *η* refers to the normalized between-class variance, computed as the ratio of the between-class variance $\eta_{b}^{2}$ and the total variance $\eta_{t}^{2}$:

(3) $$\eta =\eta_{b}^{2}/\eta_{t}^{2},$$

where

(4) $$\eta_{b}^{2}={\varpi_{1}\varpi_{2}\left(\mu_{1}-\mu_{2}\right)}^{2},$$

(5) $$\eta_{t}^{2}=\varpi_{1}\mu_{1}^{2}+\varpi_{2}\mu_{2}^{2}$$

and $\varpi_{1}$ and $\varpi_{2}$ are the weights corresponding to the two classes, while $\mu_{1}$ and $\mu_{2}$ are the means of the two Gaussian distributions [45,46]. The weights are relative proportions of pixels belonging to each class, such that their sum is always 1.

→ Metric #3: Mutual information (*M*)

Mutual information [47,48,49] is a measure of the mutual dependence between two variables. Each of the water and oil classes was considered to be a random variable, and *M* was computed according to the following formula (see [49]):

(6) $$M\left(A,B\right)=\sum_{a,b}p_{A,B}\left(a,b\right)\cdot log\frac{p_{A,B}\left(a,b\right)}{p_{A}\left(a\right)\cdot p_{B}\left(b\right)}\;,$$

where *A* and *B* are random variables, $p_{A}\left(a\right)$ and $p_{B}\left(b\right)$ are the marginal probability distributions, and $p_{A,B}\left(a,b\right)$ is the joint probability distribution. The mutual information gives an indication of how knowing one of the variables reduces the uncertainty with respect to the second variable. For two independent variables, the mutual information is zero, which means that knowing one variable gives no indication about the other variable. When the separability between classes improves, the mutual information decreases; thus, a higher *M* indicates a stronger relationship (or correlation) between the two classes. Here, a lower *M* is preferred, as it indicates better separability.

### 2.5. Special Cases

Apart from the statistical analysis, a series of special cases were also investigated individually. These cases referred to recognized issues or challenges when dealing with images taken over water surfaces: viewing angles, sky reflections, and bottom effects. The H_45R experiment (see Table 1) was used to analyze the influence of the relative viewing azimuth on the acquired oil spill images. Bottom effects identified in the acquired images were analyzed in order to estimate how they might affect automated detection algorithms. The images acquired with a viewing angle of 30° were vastly affected by sun glint; thus, they can be used to understand whether the different light reflection mechanisms specific to this situation could enhance the ability to observe the oil spills. Finally, image pairs were analyzed to observe whether clouds are potential interferers that could challenge the potential of automated algorithms in spotting oil spills over water.

## 3. Results

### 3.1. Results in Controlled Experiments

#### 3.1.1. Bimodal Gap (*G*)

Table 2 shows, for all of the experiments listed in Table 1, the relative percentage difference between the value of the bimodal gap (*G*) for a band and the best value over all bands (the highest one for this metric). The cells highlighted in grey color indicate the best performing bands, while metrics highlighted in bold indicate the bands whose relative difference from the best band is below or equal to the arbitrary limit of 10% (in absolute value).

From Table 2, it can be seen that the UV camera exhibits the highest bimodal gaps in a majority of cases (9 out of 13). There are only two cases for which the RGB camera should be preferred: U_NN and M_30. For some cases (H_30, D_NN, and D_30), the two cameras exhibit similar performance in terms of bimodal gap (*G*).

#### 3.1.2. Normalized Between-Class Variance (*η*)

Table 3 shows, for all of the experiments listed in Table 1, the relative percentage difference between the value of the normalized between-class variance (*η*) for a band and the best value over the bands (the highest one for this metric). Similarly to Table 2, the cells are colored for the best performing bands and the metric values are highlighted if the relative difference from the best band is below or equal to the arbitrary limit of 10% (in absolute value).

Table 3 shows that the UV camera also attains the highest *η* in a majority of cases (8 out of 13), followed by the R band image type, which is the best performer in terms of between-class variance for the marine diesel observations in all cases. This good performance of the R band for marine diesel might be explained by the fact that this type of oil has an apparent red color. This means that those oil pixels reflect more light around red wavelengths, leading to a clustering of their intensities around those specific wavelengths and, in turn, to higher *η* for this specific oil type. For this oil type, the UV camera is the second-best performer for all viewing angles in terms of between-class variance.

#### 3.1.3. Mutual Information (*M*)

In Table 4, the relative percentage difference between the value of the mutual information *M* for a band and the best value over the bands (the lowest one for this metric) is reported.

Similarly to the previously analyzed metrics, the UV camera also exhibits the best performance in a majority of cases (8 out of 13) for the *M* metric, followed by the blue band, which attains the lowest *M* in four experiments.

#### 3.1.4. Synthesis of Metrics 

Table 5 expands on the findings reported in Table 2, Table 3 and Table 4. For each elementary experiment, whenever a spectral band performed best in terms of one metric, that specific metric is listed in the corresponding cell. As an example, for the H_NN experiment, the UV band performed best in terms of all metrics. Metrics between brackets indicate that the difference with respect to the best value is at most 10% in absolute value. The table shows that U_NN is a singular case for which the red band outperforms the others for all computed metrics. Except for this case, the UV band performs better or comparable to the best-performing band for at least one discrimination metric. Furthermore, in 9 out of 13 experiments, the UV band performed best for at least two of the three considered metrics. The results obtained from the controlled experiments provide a first answer to the fundamental questions mentioned in the Introduction. They show that the UV camera performs better than the RGB camera for all tested oil types representative of port spills, and under nearly all tested observation conditions.

### 3.2. Results in Port Imagery Acquired from a Vessel

In order to answer to the fourth fundamental question about the applicability of the results found in controlled environments to real-world images, the metrics described in Section 2.4 were computed for the images acquired at the Port of Antwerp–Bruges from the PROGRESS vessel and analyzed in a similar way to those taken during the controlled experiments. In addition to this objective, the PROGRESS images were used to derive a methodology for automatic adaptation of the camera settings to environmental conditions.

#### 3.2.1. Metrics in Real-World Data

The UV-RGB camera system installed onboard PROGRESS captured oil spills on several days. Three dates, for which both RGB imagery and UV imagery were recorded, were analyzed: 12th March, 21st May, and 22nd May. In all cases, the selected UV imagery acquired with gain = 20 and IT = 30ms was selected. Due to the sequential image acquisition, pairs of images based on low time differences could not be determined. Instead, the time range in which the oil spill was visible in the RGB imagery was identified, and then all of the available images for the two cameras during that specific time range were selected. The (normalized) images were preprocessed such that they attained similar viewing angles (as explained in Section 2.3.2), and they were cropped to avoid the presence of the reference panel in the preprocessed images. In this way, only images containing views of the water surface (with and without oil) were obtained. Table 6 shows the numbers of RGB and UV images available for each considered date. In this table, it can be seen that there are fewer UV images available in comparison to RGB images. Due to the time gap between the image acquisitions, along with the continuous movement of the vessel in the area, the analyzed images did not cover the same area. In order to be able to apply the statistical calculus to the UV and RGB images in a fair way, rectangular regions were manually drawn to define water and oil images in a selected set of images in which this distinction was possible visually. In the end, the final number of image pairs used in the analysis was three, three, and four, respectively, for the three dates considered.

The discriminative metrics described in Section 2.4 were computed for the real-world imagery. They were in agreement with the controlled experiments and, therefore, provided the answer to the fourth fundamental question about the applicability of results obtained from the controlled experiments to the real world. Moreover, remarkably, the UV imagery achieved the best performance for all considered metrics. As shown in Table 7, the other spectral bands approached the UV performance in terms of mutual information for the last two dates. It is important to note that the vessel was in motion during the data acquisition; thus, the relative viewing azimuth could differ from one image to another. Moreover, due to waves, a fixed viewing angle cannot be ensured for this type of carrying platform. The fact that the data were acquired on different days and under different conditions with respect to the viewing angle and the Sun’s position consolidates the idea that passive UV images are strong, reliable alternatives to RGB imagery in port environments in terms of discriminative power between oil-covered and clean water areas.

#### 3.2.2. Estimation of Optimal Camera Settings

As described in Section 2.3.2, the UV camera was programmed to acquire series of images with different gains and integration times. In the long term, this strategy provides the means to derive optimal acquisition parameters and then to avoid the systematic recording of many useless images (dark/saturated). The estimation of the optimal camera settings, relying on series of images, is presented here for the UV camera, but it could be applied to series of images from any camera.

**Figure 8:**
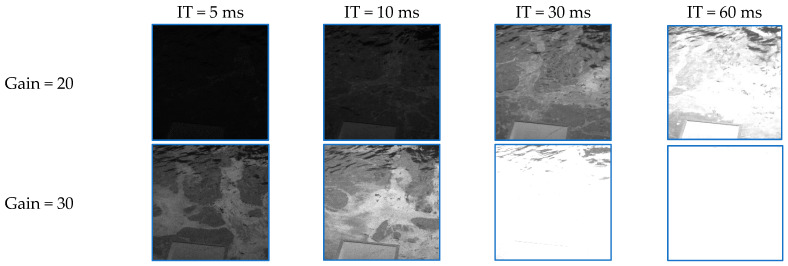
Imagery acquired with different camera settings. The bottom-right image is entirely saturated.

Figure 8 illustrates the apparent differences between images acquired with various settings. Note that the images were acquired during an oil spill, and that the quality of the images dramatically varies across the different settings.

**Figure 9:**
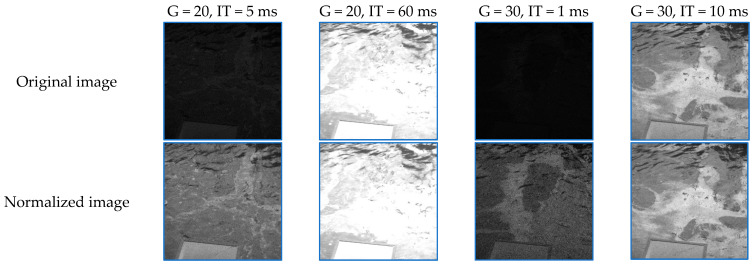
Examples of normalized images for various gains and integration times.

The image series can be seen as batches of images allowing for data quality evaluations. Lower gains and integration times are likely to be insufficient to record a good signal, while higher gains and integration times may lead to image saturation, as illustrated in Figure 8. However, the inclusion of the reference panel in the field of view allows for a normalization of the images that, at least apparently, leads to very good results. Specifically, if we divide the intensities of all pixels by (a multiple of) the mean intensity of the panel pixels and then normalize them to the 0–255 range, we obtain visually more appealing images, especially for the dark images (low gain and/or low integration time), as illustrated in Figure 9, where G represents the gain and IT represents the integration time.

**Figure 10:**
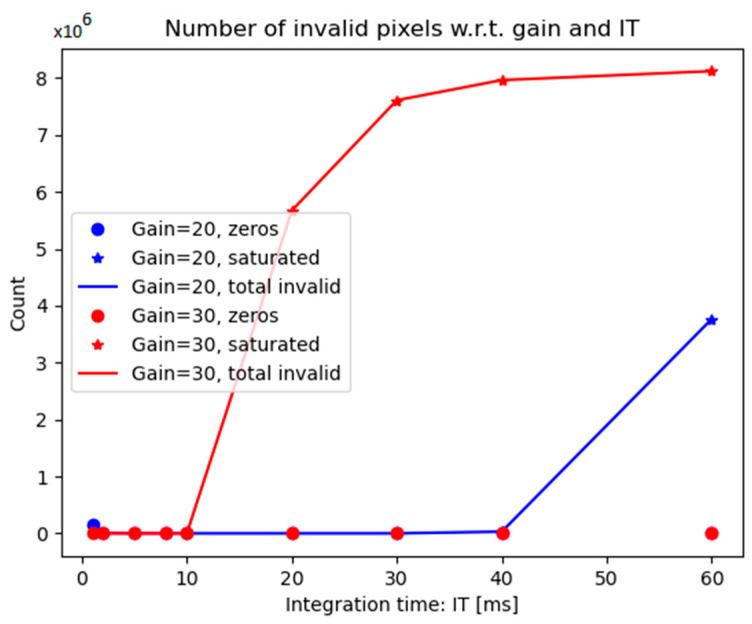
Number of invalid pixels plotted against integration time for a batch of images. The lowest values indicate optimal data acquisition settings.

Despite their better visual appearance, the normalized images still contain invalid values (black or saturated pixels). A simple inventory of these pixels could help in deriving optimal camera settings. Figure 10 plots the number of invalid pixels (zero-valued pixels represented by circles, saturated pixels represented by stars, and the total represented by connected points) for the images of a batch acquired during an oil spill on 12 March 2024. The optimal camera settings are the ones that minimize these values (and also lead to better quality metrics). In this case, a gain of 20 combined with any integration time between 5 ms and 40 ms, or a gain of 30 and integration times below 10 ms, would lead to high-quality images. These values can vary with the weather conditions and the imaged areas; thus, a long-term monitoring system such as the one used on PROGRESS allows for a more precise retrieval of these parameters, which is generally applicable in a variety of weather conditions.

Furthermore, the optimal values can be linked to weather conditions if dedicated sensors (e.g., illumination sensors) record weather parameters concomitantly. In that case, instead of computing a general set of parameters to be used at any time, adaptive parameters linked to the weather can be computed such that the camera relies on the optimal data acquisition parameters at any given moment, based on graphs like the one shown in Figure 10.

### 3.3. Special Cases

Table 2, Table 3, Table 4 and Table 5 indicate that the UV data outperform the other data types in terms of class separability for nearly all cases. In the following section, we focus on specific situations and parameters to better observe differences between the UV data and the other data types.

→ **Influence of the relative viewing azimuth**

**Figure 11:**
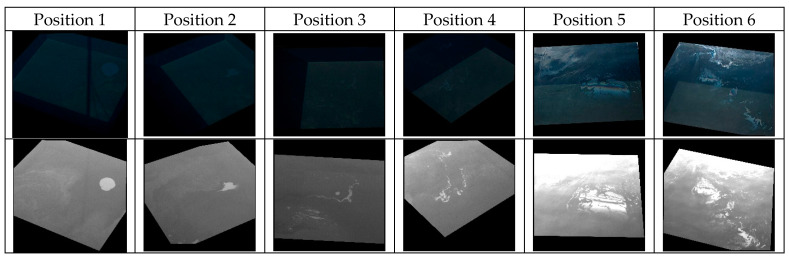
Representative images from the rotation experiment on 27 February 2024. One RGB-UV pair is shown for each of the six positions (top: RGB; bottom: UV).

The rotation experiment was specifically designed to observe how the relative viewing azimuth (the difference between the viewing azimuth and sun azimuth) influences the capabilities of the cameras to observe oil spills. Images were captured from a viewing angle of 45 degrees, from various positions (describing a sector of a circle) around the hydraulic oil container. The rotation experiment was executed in sunny weather. Six positions were used to describe a semi-circle around the container, with an angle of approximately 40 degrees in between the positions with respect to the center of the water surface. The series of measurements started with the Sun at the back of the camera and ended after completing a sector of a circle spanning approximately 200 degrees. For each position, 0.05 mL of oil was poured on the water surface. During this experiment, the RGB camera operating in automatic exposure acquired a weak signal over the water surface for the first four positions, as shown in Figure 11, where one representative pair of RGB-UV images is shown for each position. These undesired effects are also possible in a port environment, where bright objects can enter the field of view of the camera. In contrast, undesired effects (i.e., saturation) were observed in the UV imagery for the last two positions, for which the cameras were facing the Sun.

Even considering the above limitations, the images from Figure 11 show that the relative viewing azimuth angle is important for both cameras. In the particular configuration illustrated in this figure, the UV camera operating with fixed parameter settings appears to retrieve more informative data than the RGB camera operating with adaptive exposure. For further comparison, we refer to a similar experiment performed one week earlier (20 February 2024). The campaign from 20 February provided fewer qualitative data due to the suboptimal settings used for the UV camera. However, in the case of the rotation experiment, it is highly informative to compare the datasets acquired one week apart.

**Figure 12:**
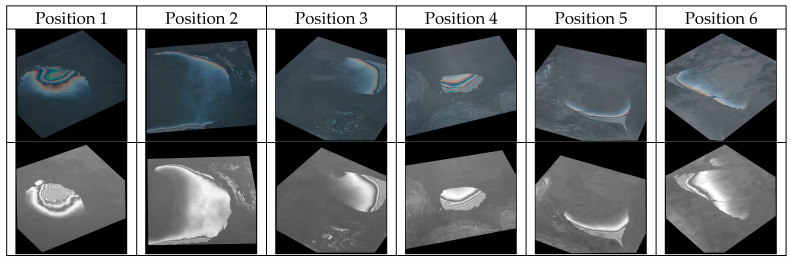
Representative images from the rotation experiment on 20 February 2024. One RGB-UV pair is shown for each of the six positions (top: RGB; bottom: UV).

Figure 12 shows pairs of RGB and UV images acquired from six different positions on 20 February 2024. It is interesting to note that the oil is easily visible in all of the RGB and UV images, from all positions. A notable difference between the two days with respect to the weather conditions is that the rotation experiment from 20 February 2024 was performed in cloudy weather, while the one on 27 February 2024 was performed in sunny weather. Thus, the images from Figure 11 and Figure 12 suggest that uniform coverage of the sky by clouds makes the relative position of the camera less critical when acquiring images, while a mixed sky or the presence of the Sun introduces additional challenges. The undesired effects shown in Figure 11 for both the RGB and UV cameras (low signal over water for the first positions and saturation for the last positions, respectively) can be alleviated by implementing strategies to adapt the camera parameters to environmental conditions, like the one described in Section 3.2.2.

→ **Bottom effects**

**Figure 13:**
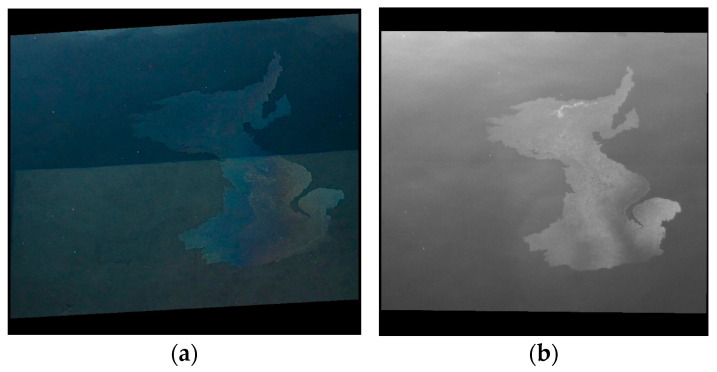
Illustration of bottom effects: (**a**) RGB image; (**b**) UV image.

In some images, the container walls (inner faces) and their shadows at the bottom of the container are visible in the RGB imagery. Figure 13 illustrates this case for the experiment D_45.

In Figure 13, it can be observed that the RGB image suffers from bottom effects, as the pixels at the top of the image are darker than the ones in the lower part of the image, due to the shadow thrown by one of the container’s lateral walls onto the container’s bottom. This is an issue for potential automated oil spill delineation methods based on pixel intensity. The UV image exhibits these effects in an almost imperceptible way, which is an advantage.

**Figure 14:**
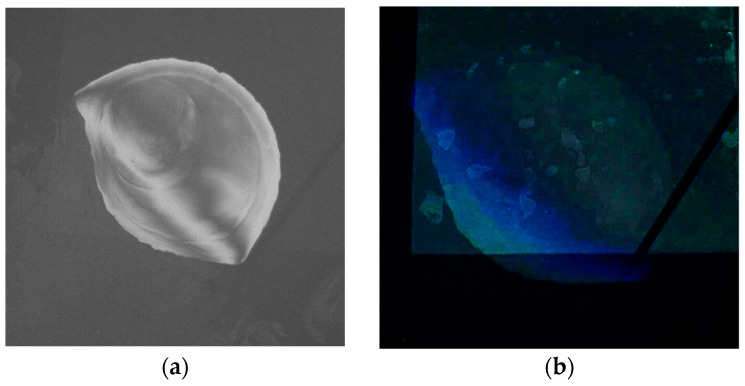

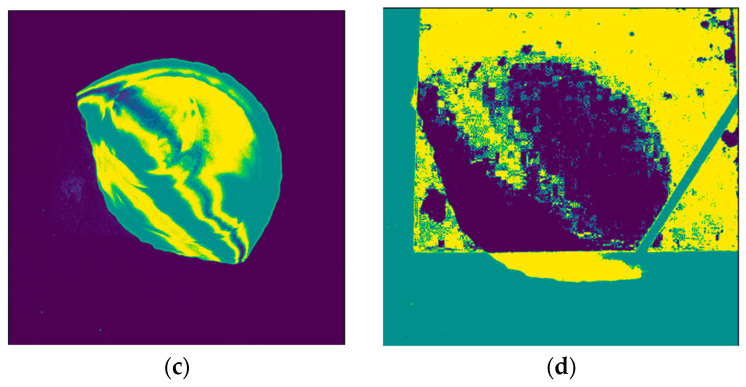
Bottom and shadow effects in RGB and UV images for a near-nadir viewing: (**a**) Example of a UV image. (**b**) Example of corresponding preprocessed RGB image. (**c**) Classification map of the UV image. (**d**) Classification map of the RGB image.

Another example, also involving shadow influence, is shown in Figure 14. The images belong to an experiment performed with an NN viewing angle. Apart from the dark parts induced by the container walls, there is also a shadow thrown by the camera’s pole onto the water surface. The RGB image becomes visually more informative after the contrast and illumination are manipulated (increased by 50%) in Figure 14b, but the limits of the oil stain are still hardly discernible. Moreover, the spatial patterns observable in the oil spill region in Figure 14b, resembling small stains, are not oil patterns but dust accumulated on the bottom of the container. They are not visible in the UV image in Figure 14a.

Figure 14c,d show classification maps of the images from Figure 14a,b, respectively. They were produced by an unsupervised k-means clustering algorithm [50], with the number of classes set to three. It can be seen that, in the RGB classification, the darker region largely forms one class, and the parts affected by the bottom effects can be easily observed due to the sharp transition between the class represented in green and the other classes. Moreover, the oil area falling on the part of the image affected by bottom effects (the lower part of the oil stain) is wrongly assigned to a water class, represented in yellow. In the same classification map, the water areas are erroneously assigned to two distinct classes, represented in green and yellow. The shadow thrown by the camera’s pole appears as an obliquely delimited area inside the yellow region, crossing both the oil and water areas. In Figure 14c, the water class is largely represented by a single class, represented in blue, and the other two classes capture the variability inside the oil stain. In this figure, the pole’s shadow does not influence the classification of the water class, and the oil area is clearly delimited.

→ **Sun glint**

**Figure 15:**
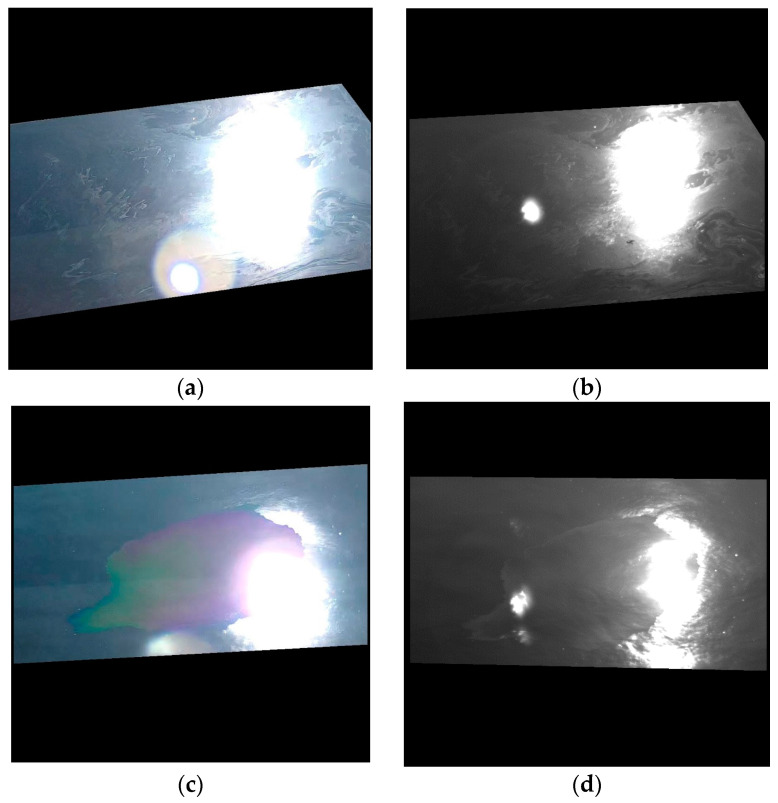
Examples of images affected by sun glint: (**a**) RGB image—hydraulic oil; (**b**) UV image—hydraulic oil; (**c**) RGB image—used oil; (**d**) UV image—used oil.

Most of the cases where the UV data were inferior to other data types in terms of metrics (especially the bimodal gap) refer to NN or 30° viewing angles. On the one hand, as mentioned earlier, the NN viewing angle is not preferred in practice, as it drastically limits the coverage area, making the monitoring more difficult in an automated system. On the other hand, the images acquired with a viewing angle of 30° were affected by sun glint due to the relative position of the camera with respect to the Sun. In the metrics’ computation, saturated pixels and dark pixels (i.e., with DN values of 255 or 0, respectively) were neglected, but the sun glint also induced high DN values around the saturated area, which might have affected the accuracy of the computed metrics. In Figure 15, examples of images affected by sun glint are shown. Note that oil features are visible around the saturated areas for both image types.

→ **Clouds**

**Figure 16:**
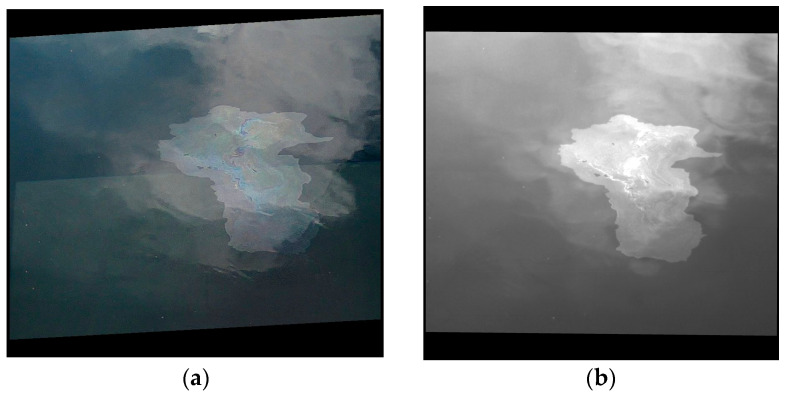

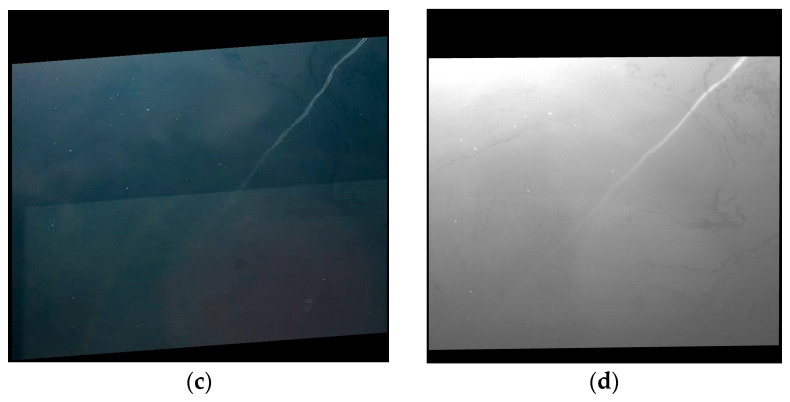
Examples of images affected by clouds and other undesired reflections: (**a**) RGB image affected by clouds; (**b**) UV image affected by clouds; (**c**) RGB image capturing the contrails of an airplane; (**d**) UV image capturing the contrails of an airplane.

Empirical observations (by visual inspection) of the acquired images showed that both the UV and RGB images captured clouds reflected by the scanned surface. An example is shown in Figure 16a,b. The same undesired effects can also be seen in Figure 4. Apart from clouds, other undesired objects or phenomena can also be spotted in the images, as illustrated in Figure 16c,d, where the contrails left by an airplane are visible in both the RGB and UV images. It can be concluded that the UV images are not free from cloud/atmospheric reflections, similar to the RGB images.

The special cases show that both the UV and RGB imagery suffer from undesired effects such as sun glint and cloud reflections. The relative viewing azimuth angle impacts the visibility of oil in RGB images more than in UV images in sunny weather; thus, its influence is weather-dependent. However, this observation needs further experimental confirmation under similar exposure modes for the two imaging systems, e.g., by implementing the automatic camera settings adaptation procedure for both cameras before computing the discrimination metrics. The main finding in the analysis of the special cases concerns the impact of the bottom effects, which is significantly lower in the UV imagery, showing a clear advantage of this camera in comparison to the RGB camera. This subsection completes the answers to the four fundamental questions, considering more viewing and environmental conditions, and highlighting another advantage of the UV camera.

## 4. Discussion

The experiments designed in this study addressed the particular case of oil spills in port environments. The need for such a study stems from the particularities of ports in comparison to open-sea environments, as well as the difficulties in applying established surveillance and detection methods in the open sea. The limitations can be summarized as follows: (i) Ports are crowded environments where free satellite imagery, including optical and SAR images provided by the Sentinel-2 and Sentinel-1 satellites, does not have a sufficiently high spatial resolution to resolve all objects; moreover, high-resolution satellite images offered by commercial companies induce additional costs and, similar to free-access data, do not provide a continuous monitoring of the port. (ii) Active sensors, such as those exploiting the fluorescence of oil compounds, are relatively heavy and voluminous, inducing additional safety and security risks if used in ports. (iii) The oil spilled in ports is usually refined and, therefore, different from the crude oil in open seas, leading to a need to assess the applicability of the established methods in the case of ports. (iv) Other sensing solutions, such as SWIR cameras, are more efficient for thick oil layers than for thin oil slicks, which is the case at ports. (v) The risks to human health are higher at ports than in the open sea, due to the presence of people in the area; thus, the intervention procedures are different, and continuous monitoring is needed. Therefore, lightweight systems, easily deployable in operational setups, are encouraged.

The controlled experiments designed in this study showed that the UV camera has higher discriminative power between the oil and water classes in almost all cases. As shown in Table 5, which expands upon Table 2, Table 3 and Table 4 by synthesizing the discriminative power of each band in terms of the various metrics, there is only one case where the UV band is inferior to another band: the near-nadir viewing of used oil. Unlike the other cases, the used oil might contain not only oil but also nano- or microparticles that result from the movement of the lubricated metallic parts and other residuals, e.g., from fuel or oil burns. Thus, the fact that the red band outperforms the other bands might be due to the presence of these non-oil components, not to the oil itself. However, as an overall observation, the near-nadir viewing case appears to be the only one where the differences between bands are reduced for diesel and marine diesel. This viewing angle is impractical in port environments, as the covered area is restricted in comparison to oblique viewing angles. The near-nadir view is not ideal for the surveillance of large areas; thus, the other viewing angles, where the UV camera outperforms the RGB camera, are more representative for operational setups. Equally importantly, the UV camera showed much less sensitivity to bottom effects. This is important if tailored algorithms are designed for the automated delineation of the oil spills and other classes are considered in the analysis, which was beyond the scope of the current study. For example, seagrass might be observed by an RGB camera but not by a UV camera, and the same holds for underwater plumes. A downside that equally affects the UV and the RGB images is the presence of clouds and other undesired reflections, which are captured by both data types, constituting an obstacle for automated algorithms due to the confusion that they introduce between classes. When analyzing images affected by sun glint, the oil can be spotted around the saturated areas in both UV and RGB images. However, for the UV camera, this is not necessarily an advantageous situation, as was shown to be the case for satellite data [18,51,52], where other physical phenomena prevail, since the oil can be spotted from all tested positions. In practice, the data acquisition platforms (drones and/or ships) will scan the water surface while continuously changing position; thus, the images affected by sun glint could be discarded from the analysis without significantly decreasing the chances of spotting the polluted area.

The two so-called rotation experiments, in which the cameras acquired images from a fixed viewing angle but with different relative viewing azimuth angles, showed that the UV camera is not significantly dependent on illumination angles, disregarding sun glint cases. In the reported experimental setup, the environmental illumination impacted the visibility of the spills in the acquired imagery much less, unlike the RGB images, where the presence of the Sun strongly impacted the visibility of the oil, depending on the relative viewing azimuth, as shown in Figure 11 and Figure 12. In practical terms, this means that, in order to acquire useful images of the spills, the RGB camera has stronger restrictions with respect to observation angles than the UV camera, which is another advantage for the latter. However, before claiming that this observation is generally valid (irrespective of the acquisition setup), further investigations are needed, e.g., when automated adaptation of camera settings is used concomitantly for both systems.

The development of automated oil spill delineation algorithms is beyond the scope of this paper, which focused on the fundamental capabilities of different spectral bands in discriminating between two classes of interest: clean water, and water covered by oil. However, as shown in Figure 14, even simple approaches such as the k-means clustering algorithm prove that undesired bottom effects can impede the correct delineation of the areas polluted by oil, and to a much greater extent in RGB images than in UV images.

The data acquired in real-world conditions at the Port of Antwerp–Bruges confirmed the observations made in the controlled experiments. It is remarkable that, in the port imagery, the UV camera achieved the best performance with respect to all computed metrics. The data acquisition setup on the PROGRESS vessel allowed for inferring ideal camera settings, either fixed for all types of weather or tailored to instantaneous conditions by plugging in weather parameters recorded by dedicated sensors. In operational setups, the procedure can be progressively refined once more data are available. In the case of oil spill detection and monitoring with drones, imagery of a reference target when the drone leaves its parking area could also provide information for choosing the right camera settings. By using optimal camera settings during data acquisition, automated algorithms dedicated to the detection of the oil spills can ingest images with a good dynamic range for water and oil, therefore ensuring optimal efficiency. Overall, the UV cameras appear to be a viable solution for detecting and monitoring oil spills in port environments. Nevertheless, an interesting path to explore is the discriminative power of the UV camera when changing the UV spectral band, particularly by analyzing shorter wavelengths.

## 5. Conclusions

In this paper, the capabilities of UV cameras to detect and monitor oil spills in port environments were evaluated. This special type of oil spills has not been studied extensively in the previous literature, despite their common occurrence. The specificities of oil spills in ports call for tailored data types and methods, as many of the established methods applicable to crude oil spills in the open sea are not applicable (e.g., SWIR data have limited capabilities for thin oil films). In ports, additional constraints call for lightweight surveillance solutions; thus, this study can be considered to be one of the first to prove the suitability of such solutions. Our study provides insights into the capabilities of different spectral bands to distinguish between water and oil surfaces by computing separability metrics for various refined oil types, in both controlled and real-world data acquisitions.

The experiments carried out in a controlled environment showed that the UV camera is superior to RGB cameras in terms of discriminative power when two targeted classes are considered: oil surfaces, and clean water surfaces. These experiments were designed to cover a variety of settings, some of them being under the control of the operators (camera settings, relative viewing azimuth angles and positions, oil type and quantity) and others not being controllable by the operators (weather conditions). The discriminative metrics computed for the UV and RGB cameras show that the UV camera is a strong alternative to RGB cameras in port environments. Furthermore, the analysis of real-world images acquired at the Port of Antwerp–Bruges from a mobile platform (a vessel) confirmed the conclusions drawn from the controlled experiments. In this setup, a methodology to infer optimal camera settings with respect to instantaneous weather conditions was also defined.

This work opens a wide range of future research opportunities. While our study proves that the UV camera has a high discriminative power between water and oil areas, other classes of materials were not considered. In ports, it is expected that various objects will often enter the field of view of the cameras, so it is important to explore whether and how they can be separated from the classes of interest. The water’s constituents can also influence the discrimination power, and further research is needed to determine how other phenomena, such as the occurrence of turbidity plumes or phytoplankton blooms, impact the class discrimination. It is possible that using at least one other data type together with the UV data could be helpful to achieve this goal. For example, it is expected that technological advancements will support the continuous monitoring of the port basin by SAR sensors and other system types, such that the advantages of these systems, e.g., the lack of sensitivity to the presence of clouds, could be exploited in conjunction with optical sensors similar to the ones used in our study for an accurate representation of the port configuration at any given moment, including pollution events like oil spills. Furthermore, automated algorithms are needed in order to delimit the oil spills when they occur. When operated from drones, the flight parameters can impact the data quality; thus, it is of interest to study optimal ways to operate these platforms while considering the highest norms of safety for people in the area.

Our study provides a positive answer to a fundamental question: do UV cameras bring advantages over RGB cameras in terms of discrimination power between water and refined oil areas in ports? Thus, this study constitutes a first building block towards an operational monitoring system that includes UV cameras for oil spill detection in port environments, which will be a subject of our future work.

## Figures and Tables

**Table 1:** Experiments performed on 27 February 2024.

Oil Type	Identifier	Viewing Angle	Observations
H (hydraulic)	H_NN	NN	Cloudy, regular wind
	H_45	45	Sunny, mild wind
	H_45R	45_Rotation	Sunny, mild wind
	H_30	30	Sunny, mild wind, sun glint
U (used)	U_NN	NN	Cloudy, regular wind
	U_45	45	Sunny, mild wind
	U_30	30	Sunny, mild wind, sun glint
D (diesel)	D_NN	NN	Cloudy, regular wind
	D_45	45	Mixed sky, mild wind
	D_30	30	Sunny, mild wind, sun glint
M (marine diesel)	M_NN	NN	Cloudy, regular wind
	M_45	45	Sunny, mild wind
	M_30	30	Sunny, mild wind, sun glint

**Table 2:** Relative percentage difference values for bimodal gap *G* computed for each performed experiment. The grey cells indicate the best performing bands, and values highlighted in bold indicate bands whose performance is close to the best one.

Identifier	Image Type (Spectral Band/Transformation)				
	UV	Blue	Green	Red	Gray
H_NN	**0**	−21	−36	−46	−37
H_45	**0**	−85	−91	−93	−91
H_45R	**0**	−81	−87	−95	−88
H_30	**−6**	**0**	**−3**	**−3**	**−2**
U_NN	−29	−39	−21	**0**	−17
U_45	**0**	−77	−66	−73	−69
U_30	**0**	−23	−20	−31	−23
D_NN	**−6**	**−5**	**0**	**−9**	**−3**
D_45	**0**	−43	−39	−41	−40
D_30	**0**	**−2**	**0**	**−10**	**−2**
M_NN	**0**	−37	−75	−59	−77
M_45	**0**	−65	−81	−86	−83
M_30	−25	**0**	**−4**	**−11**	**−5**

**Table 3:** Relative percentage difference for normalized between-class variance (*η*) computed for each performed experiment. The grey cells indicate the best performing bands, and values highlighted in bold indicate bands whose performance is close to the best one.

Identifier	Image Type (Spectral Band/Transformation)				
	UV	Blue	Green	Red	Gray
H_NN	**0**	−42	−50	−46	−46
H_45	**0**	−74	−83	−39	−78
H_45R	**0**	−71	−86	−90	−81
H_30	**0**	−43	−35	−16	−32
U_NN	−79	−63	−37	**0**	−31
U_45	**0**	−69	−38	−28	−41
U_30	**0**	−67	−53	−41	−51
D_NN	−75	−42	−17	**0**	−17
D_45	**0**	−31	−19	−17	−21
D_30	**0**	−70	−61	−48	−58
M_NN	−38	−58	−88	**0**	−88
M_45	−35	−60	−77	**0**	−74
M_30	**−3**	−36	−26	**0**	−23

**Table 4:** Relative percentage difference for mutual information (*M*) computed for each performed experiment. The grey cells indicate the best performing bands, and values highlighted in bold indicate bands whose performance is close to the best one.

Identifier	Image Type (Spectral Band/Transformation)				
	UV	Blue	Green	Red	Gray
H_NN	**0**	19	20	20	19
H_45	60	**0**	30	70	30
H_45R	100	**0**	32	80	40
H_30	**0**	44	81	125	88
U_NN	178	35	18	**0**	15
U_45	**0**	70	24	57	30
U_30	**0**	186	268	355	223
D_NN	263	**0**	20	70	27
D_45	**0**	34	31	53	25
D_30	**0**	34	26	47	26
M_NN	29	**0**	28	35	25
M_45	**0**	42	119	119	115
M_30	**0**	25	34	47	19

**Table 5:** Overview of the best-performing data types (bands) for each performed experiment. One cell is highlighted in grey if the corresponding band had the best or near-best performance for a larger number of metrics than the other bands.

Identifier	Image Type (Spectral Band/Transformation)				
	UV	Blue	Green	Red	Gray
H_NN	*G*, *η*, *M*				
H_45	*G*, *η*	*M*			
H_45R	*G*, *η*	*M*			
H_30	(*G*), *η*, *M*	*G*	(*G*)	(*G*)	(*G*)
U_NN				*G, η, M*	
U_45	*G*, *η*, *M*				
U_30	*G*, *η*, *M*				
D_NN	*(G)*	(*G*), *M*	*G*	(*G*), *η*	(*G*)
D_45	*G*, *η*, *M*				
D_30	*G*, *η*, *M*	(*G*)	(*G*)	(*G*)	(*G*)
M_NN	*G*	*M*		*η*	
M_45	*G*, *M*			*η*	
M_30	*(η*), *M*	*G*	*(G)*	*η*	(*G*)

**Table 6:** Numbers of available and used images for the three considered dates.

Date	Time Range	#RGB Images	#UV Images	#Image Pairs Used
12 March 2024	13:16:46–14:33:05	12	5	3
21 May 2024	14:01:51–16:17:50	18	8	3
22 May 2024	10:02:03–12:16:38	19	9	4

**Table 7:** Discriminative metrics computed for real-world imagery. The grey cells indicate the best performing bands, and values highlighted in bold indicate bands whose performance is close to the best one.

Metric	Date	Image Type (Spectral Band/Transformation)				
		UV	Blue	Green	Red	Gray
*G*	12 March 2024	**0**	33	49	52	48
	21 May 2024	**0**	**2**	23	32	23
	22 May 2024	**0**	17	38	49	39
*η*	12 March 2024	**0**	−82	−89	−89	−88
	21 May 2024	**0**	−56	−73	−75	−72
	22 May 2024	**0**	−85	−91	−94	−92
*M*	12 March 2024	**0**	34	31	25	46
	21 May 2024	**0**	−18	**−4**	**2**	**2**
	22 May 2024	**0**	**−5**	**−10**	**−7**	**8**

## Data Availability

The data used in this paper are not publicly available.
